# Supplementary material for: NanoDefiner e-Tool: An Implemented Decision Support Framework for Nanomaterial Identification
Source: Materials (Basel). 2019 Oct 4;12(19):3247. doi: 10.3390/ma12193247 (PMC6803960; doi:10.3390/ma12193247)
Supplement: Supplementary file 1 [file materials-12-03247-s001.pdf]

Supplementary Materials: NanoDefiner e-Tool Knowledge Base Attributes

Raphael Brüngel, Johannes Rückert, Wendel Wohlleben, Frank Babick, Antoine Ghanem, Claire Gaillard, Agnieszka Mech, Hubert Rauscher, Vasile-Dan Hodoroaba, Stefan Weigel and Christoph M. Friedrich

Measurement technique (MT) recommendation for a specific material uses attributes, representing structured and formalized knowledge. For particle type description attributes listed in Table S1 are used to describe its physicochemical properties. Likewise, an MT is described via attributes listed in Table S2 (material-related properties), Table S3 (performance-related properties), and Table S4 (technical and economic properties). For MT recommendation not all MT attributes are matched.

Table S1. Matchable attributes for particle type description.

Attribute Name	Attribute description	Matched
`trade_form`	Trade form of particle type	Yes
`dispersibility`	Dispersibility of particle type by protocols	Yes
`chemical_composition`	Chemical compositions of particle type	Yes
`composites`	Composites of particle type	Yes
`dimensions`	Particle small dimensions number of particle type	Yes
`shape`	Particle shapes of particle type	Yes
`analysis_temperature`	Stable analysis temperature range of particle type (°C)	Yes
`electron_beam`	Particle type degradation under electron beam radiation	Yes
`polydispersity`	Grade of polydispersity in particle type	Yes
`multimodality`	Grade of multimodality in particle type	Yes
`conductivity`	Conductive properties of particle type	Yes
`magnetism`	Magnetic properties of particle type	Yes
`functionalisation`	Functionalisation of particle type	Yes
`light_absorption`	Light absorbing properties of particle type	Yes
`fluorescence`	Fluorescent properties of particle type	Yes
`aggregation`	Particle aggregation in particle type	Yes
`agglomeration`	Particle agglomeration in particle type	Yes
`release_iam`	Release of ions, atoms, and molecules of particle type	Yes
`wr_size_range`	Particle size range (nm) in particle type	Yes
`presence`	Presence of different sized subnano (diameter $<$ 1 nm in all dimensions) or susnano (diameter $>$ 100 nm in all dimensions) particles in particle type	Yes
`os_vacuum`	Degradation of particle type under vacuum	Yes

Table S2. Matchable material-related attributes for measurement technique description.

Attribute Name	Attribute description	Matched
`trade_form`	Material trade forms supported by MT	Yes
`preparation`	Suitable SOP-based preparation techniques for MT	No [a]
`dispersion_medium`	Dispersion media supported by MT	No [a]
`dispersibility`	Dispersibilities by protocols supported by MT	Yes
`chemical_composition`	Chemical compositions supported by MT	Yes
`composites`	Composites supported by MT	Yes
`dimensions`	Number of small dimensions supported by MT	Yes
`shape`	Particle shapes supported by MT	Yes
`analysis_temperature`	Analysis temperature range (celsius) of MT	Yes
`electron_beam`	Use of electron beam by MT	Yes
`polydispersity`	Grades of polydispersity supported by MT	Yes
`multimodality`	Grades of multimodality supported by MT	Yes
`conductivity`	Conductive properties supported by MT	Yes
`magnetism`	Magnetic properties supported by MT	Yes
`functionalisation`	Functionalization supported by MT	Yes
`light_absorption`	Light absorbtion supported by MT	Yes
`fluorescence`	Fluorescence supported by MT	Yes
`aggregation`	Aggregation supported by MT	Yes
`agglomeration`	Agglomeration supported by MT	Yes
`release_iam`	Release of ions, atoms, and molecules supported by MT	Yes

[a] Not matched for MT recommendation, but takes effect on parts of the categorization workflow.

Table S3. Matchable performance criteria-related attributes for measurement technique description.

**Attribute name**	**Attribute description**	**Matched**
`tier`	Screening (tier 1) or confirmatory (tier 2) MT	No [a]
`purpose`	MT usable for specific purpose(s)/regulations	No [a]
`disclaimer`	MT disclaimer and important information for use	No [a]
`multiconstituent`	MT supports multi-type samples	No [a]
`specific_technique`	MT provides specific techniques	No
`wr_size_range`	Particle size range (nm) measured properly by MT	Yes
`wr_concentration_range_type`	Concentration range type specified by attribute `wr_concentration_range`	No
`wr_concentration_range`	Interpretation is dependent on attribute `wr_concentration_range_type`	No
`wr_minimum_sample_type`	Sample type specified by attribute `wr_minimum_sample_type`	No
`wr_minimum_sample`	Interpretation is dependent on attribute `wr_minimum_sample_type`	No
`wr_linear_proportional_size`	MT's results linear/proportional with respect to particle size	No
`wr_linear_proportional_concentration`	MT's results linear/proportional with respect to particle concentration	No
`wr_detection_quantification_limit`	Size range limits (nm-scaled) of particles MT measures properly	No
`wr_sensitivity`	Trust/usability/quality level regarding MT sensitivity	No
`trueness`	Trust/use/quality level regarding MT trueness	No
`weighting_trueness`	Trust/use/quality level regarding MT trueness in weighting size fractions	No
`robustness`	Trust/use/quality level regarding MT robustness	No
`precision_type`	Precision range type specified by attribute `precision`	No
`precision`	MT precision (nm-scaled)	No
`resolution_type`	Resolution range type specified by attribute `resolution`	No
`resolution`	MT resolution (nm-scaled)	No
`size_distribution`	MT supports measurement of size distribution	No
`presence`	MT supports presence of different sized subnano ($d < 1$ nm in all dimensions) or susnano ($d > 100$ nm in all dimensions) particles	Yes
`sl_discr_nnp_same`	MT supports the discrimination from non-nanoparticles of the same composition	No
`sl_discr_nnp_same_robustness`	MT provides robustness against different types of impurities in case of discrimination from non-nanoparticles of the same composition	No
`sl_discr_nnp_diff`	MT supports discrimination from non-nanoparticles of another composition	No
`sl_discr_nnp_diff_robustness`	MT provides robustness against different types of impurities in case of discrimination from non-nanoparticles of another composition	No
`sl_discr_np_diff`	MT supports discrimination from nanoparticles of another composition	No
`sl_discr_np_diff_robustness`	MT provides robustness against different types of impurities in case of discrimination from nanoparticles of another composition	No
`measurement_aggr`	MT supports particle measurement within aggregates	No
`measurement_aggl`	MT supports particle measurement within agglomerates	No
`measurement_individual_particle`	MT supports single particle measurement in size and number	No
`constituent_particle_counting_aggr`	MT supports particle measurement in aggregates	No
`constituent_particle_counting_aggl`	MT supports particle measurement in agglomerates	No
`composition`	MT provides information on the chemical composition	No
`size_type`	MT measurands (equivalent diameters, nm-scaled)	No
`data_format`	Analysis file data formats provided by the MT	No [a]
`destructive`	Sample destruction caused by the MT	No
`os_vacuum`	Demand of vacuum by the MT	Yes
`os_sample_support`	MT demands preparation of suited supports	No

[a] Not matched for MT recommendation, but takes effect on parts of the categorization workflow.

Table S4. Matchable technical and economic attributes for measurement technique description.

Attribute	Description	Matched
direct_counting	MT support of yielding directly number-weighted size distribution	No
convertibility	MT conversion algorithm's quality	No
full_size_range	MT support of accessing the full size range according to EC NM definition	No
upper_size	MT approximate upper size limit	No
lower_size	MT approximate lower size limit (close to 1 nm)	No
smallest_particle_dimension	MT support of accessing smallest particle dimension	No
primary_particle_access_aggr	MT support of accessing primary particles within aggregates	No
primary_particle_access_aggl	MT support of accessing primary particles within agglomerates	No
availability	MT availability and wide use	No
standard	MT is a standard	No
reference	MT potential as reference method	No
eot_preparation	MT sample preparation time	No
eot_measurement	MT measurement time	No
eot_reduction	MT data reduction time	No
eot_total	MT total time until results	No
cost_instrument	MT instrument costs	No
cost_analysis	MT analysis costs	No
cost_efficiency	MT cost efficiency	No
expertise	MT required expertise	No
hyphenation	MT ability to be hyphenated to other methods.	No
vacuum	MT demand for vacuum intensity	No
sample_support	MT demand for preparation on suited supports	No
degradation	MT risk of chemical sample degradation during measurement	No
